# Improving deep learning performance for predicting large-scale geological ${{CO}_{2}}$ sequestration modeling through feature coarsening

**DOI:** 10.1038/s41598-022-24774-6

**Published:** 2022-11-30

**Authors:** Bicheng Yan, Dylan Robert Harp, Bailian Chen, Rajesh J. Pawar

**Affiliations:** 1 King Abdullah University of Science and Technology, Thuwal, Saudi Arabia 23955; 2 Earth and Environmental Sciences, Los Alamos National Laboratory, Los Alamos, NM 87544 USA

**Keywords:** Hydrology, Energy science and technology, Engineering, Mathematics and computing

## Abstract

Physics-based reservoir simulation for fluid flow in porous media is a numerical simulation method to predict the temporal-spatial patterns of state variables (e.g. pressure *p*) in porous media, and usually requires prohibitively high computational expense due to its non-linearity and the large number of degrees of freedom (DoF). This work describes a deep learning (DL) workflow to predict the pressure evolution as fluid flows in large-scale 3-dimensional(3D) heterogeneous porous media. In particular, we develop an efficient feature coarsening technique to extract the most representative information and perform the training and prediction of DL at the coarse scale, and further recover the resolution at the fine scale by spatial interpolation. We validate the DL approach to predict pressure field against physics-based simulation data for a field-scale 3D geologic $CO_2$ sequestration reservoir model. We evaluate the impact of feature coarsening on DL performance, and observe that the feature coarsening not only decreases the training time by $>74\%$ and reduces the memory consumption by $>75\%$, but also maintains temporal error $0.63\%$ on average. Besides, the DL workflow provides predictive efficiency with 1406 times speedup compared to physics-based numerical simulation. The key findings from this research significantly improve the training and prediction efficiency of deep learning model to deal with large-scale heterogeneous reservoir models, and thus it can also be further applied to accelerate workflows of history matching and reservoir optimization for close-loop reservoir management.

## Introduction

Many geologic storage^1–3^ and energy extraction processes^4,5^ can be described by fluid flow in porous media. So far the reservoir management of these processes, e.g., history matching^6^, well placement optimization^7^ and well schedule optimization^8^, heavily relies on physics-based reservoir simulation. Based on certain assumptions, physics-based reservoir simulation is capable of characterizing the complexities of physics about what we observe in lab experiments or field data. The governing equations of fluid flow in porous media are represented as partial differential equations (PDEs), and the spatial-temporal evolution of the unknown state variables (e.g., pressure *p* and saturation *S*) can be solved by traditional numerical methods such as finite difference or finite volume methods^9,10^. Nevertheless, as we perform history matching or reservoir optimization, physics-based reservoir simulation models often suffer prohibitively high computational expense, resulting from the high resolution of the geological model, the non-linearity due to heterogeneities^10^, complex fluid phase behavior^11^, and coupled physics processes such as thermo-hydro-mechanical (THM) modeling^12,13^.

In recent years, deep learning (DL) gains remarkable advances and application in the science and engineering community, with its capability to process high dimensional data^14^ and approximate continuous functions^15^. In the domain of fluid flow in porous media, recent research has focused on enhancing the capability to predict the evolution of state variables in porous media with DL. A family of physics-informed neural network (PINN) models imposes certain forms of governing equations to regularize the loss function during the training process through automatic or numerical differentiation^16–19^. These approaches ensure that the neural networks are consistent with the governing physics of fluid flow in porous media through regularizing the training processes with physics constrains. While PINN is suitable for predicting processes governed by physics with medium complexity, its computation cost may become expensive to solve problems with high dimension or high nonlinearity. Alternatively, image-based approaches have also been investigated to predict fluid flow in porous media, and mainly leverage convolutional neural networks (CNN) to approximate the nonlinear relationships between geological property maps (e.g., permeability) and fluid flow maps (e.g., pressure) in porous media^20–24^. With sparse connectivity between input and output, image-based approaches tend to be more appropriate to deal with medium scale heterogeneous porous media.

In order to effectively capture the heterogeneity with high resolution, the size of a realistic geological model can easily reach to millions of grid cells, which is likely to bring significant CPU cost and memory consumption to train deep learning models and predict the temporal and spatial evolution of state variables. Since many previous research studies^20–24^ mainly focus on small to median-size reservoir models, while limited work has been done to systematically evaluate the memory and CPU performance of DL models when predicting the evolution of state variables in such large heterogeneous reservoir models. We highlight that this becomes a potential major challenge and research gap when we need to apply deep learning methods to accelerate the prediction of large-scale reservoir models for Geological $CO_2$ sequestration or other subsurface flow and transport processes.

In this work, we describe a DL workflow to predict the evolution of pressure due to fluid flow in large-scale 3D geological models. This workflow falls into the category of image-based approaches, as it takes full advantage of CNN’s spatial topology predictive capability^25,26^, specifically Fourier Neural Operator (FNO)^24,27^, which excels in predicting physics-based processes by replacing the kernel integral operator via a convolutional operator defined in Fourier space. Compared to our previous work^24^, our main contribution in this work lies in two aspects. First, we successfully develop a feature coarsening technique to enable the FNO model to handle large-scale geological models with more than $10^6$ grid cells. This is achieved by extracting the most representative information from the input maps (e.g., permeability and porosity), and perform the training and prediction of FNO at the coarse scale. This can significantly decrease the memory consumption of the training data and computational cost for training, and thus makes DL more affordable for large scale geological models. We remark that in our previous work^24^, the reservoir model was in relatively median size with $10^4$ grid cells, and FNO can efficiently deal with such a model without the need of feature coarsening. Second, using the principle of pressure continuity, we further recover the resolution of predicted pressure fields at the fine scale through spatial interpolation. This technique is expected to be applicable to state variables that have a relatively global and continuous distribution in the reservoir domain. To validate its predictive performance, we apply the DL workflow, which is trained from physics-based simulation data, to predict the pressure evolution in a field-scale 3D heterogeneous geological $CO_2$ sequestration reservoir model. We perform a comprehensive analysis of the DL workflow to assess its memory efficiency, training efficiency and predictive accuracy.

The manuscript is structured as below. In Sect. 2, we take the geological $CO_2$ sequestration process as an example to introduce the physics governing equation of multi-phase flow in porous media. In Sect. 3, we provide the methodology of our deep learning approach, including feature selection and assembly, feature coarsening, architecture of deep neural network and the method to recover fine scale resolution of state variable. In Sect.on 4, we illustrate our numerical results to validate the efficacy and accuracy of the DL methodology. In Sect. 5, we conclude with a few remarks.

## Physics problem formulation

At a geologic $CO_2$ sequestration site where $CO_2$ is injected into a saline aquifer, the fluid phases include an aqueous phase (*a*) and a supercritical $CO_2$ phase (SC-$CO_2$), with water ($H_2O$) and $CO_2$ as the primary components in the aqueous and SC-$CO_2$ phases, respectively. The flow and transport of each component in the porous media is governed by their respective mass(material) balance equations, shown as Eq. (1),

1 $$\begin{aligned} \dfrac{\partial (\phi \sum _{\alpha } x_{\alpha ,i} {\rho }_{\alpha } S_{\alpha } ) }{\partial t} - \nabla \cdot (K \sum _{\alpha } {{\frac{{x_{\alpha ,i} {\rho }_{\alpha } k_{r,\alpha }}}{\mu _{\alpha }}} (\nabla p_{\alpha } - {\rho }_{\alpha } {\mathbf {g}} {\nabla } Z) } ) + \sum _l{\sum _{\alpha } ({\rho }_{\alpha } x_{\alpha ,i} q_{\alpha }})_l = 0 \end{aligned}$$

where subscript *i* indicates the primary component, including $H_2O$ and $CO_2$; $\alpha$ indicates the fluid phase, including the aqueous phase (*a*) and the supercritical $CO_2$ phase (SC-$CO_2$); *t* denotes time; $\phi$ is the rock porosity; $S_{\alpha }$ is the phase saturation; ${\rho }_{\alpha }$ is the fluid phase density; $x_{\alpha ,i}$ is the mole fraction of component *i* in fluid phase $\alpha$; *K* is the permeability of porous media; $k_{r,\alpha }$ is the phase relative permeability; $\mu _{\alpha }$ is the phase viscosity; $p_{\alpha }$ is the phase pressure; ${\mathbf {g}}$ is the acceleration due to gravity; *Z* is depth; $q_{\alpha }$ denotes the rate for extracting or injecting fluid phase $\alpha$ through well perforation *l*.

In Eq. (1), the first term is the fluid accumulation, the second is the advective flux based on Darcy’s law, and the third is the source or sink term. Equation (1) is constrained by several auxiliary relationships, including the equality between total fluid volumes and pore volume to ensure the volumetric balance^28^, capillary pressure constraint to relate wetting phase pressure (*a*) with non-wetting phase pressure (SC-$CO_2$), and fluid thermodynamics equilibrium to calculate fluid phase properties ($x_{\alpha ,i}$, $\rho _{\alpha }$ and $\mu _{\alpha }$). Additional details can be found in previous literatures^9,28^. In a physics-based reservoir simulator, Eq. (1) along with the auxiliary relationships are solved iteratively to predict the state variables.

## Methodology

The aim of the DL workflow is to predict the temporal-spatial evolution of pressures in a 3-dimensional(3D) porous media reservoir, providing a computationally efficient alternative to physics-based reservoir simulators. In this section, we illustrate the details of the DL workflow including feature selection and assembly, feature coarsening, deep neural network architecture, training, prediction and fine-scale resolution recovery.

### Feature selection

Here, the features are the input variables to predict the evolution of pressure as fluid flows in porous media. To be consistent with the data structure of the 2-dimensional (2D) convolutional neural network architecture we apply in this work, the features are also assembled as 2D images.

Based on the theory of reservoir simulation^28^, rock permeability (*K*) is a type of connection-based variable and contributes to the flux term (second term) in Eq. (1). It characterizes the degree of spatial connectivity of fluid flow in horizontal and vertical directions. Since the vertical permeability $K_V$ is typically significantly lower than the horizontal permeability $K_H$, e.g., we use $K_V/K_H =0.1$ in our work, the vertical connectivity contributes much less to the fluid flow than the horizontal connectivity. Therefore, we ignore the impact of vertical connectivity and slice the 3D permeability volume into 2D horizontal layer-wise images. When the impact of vertical connectivity on pressure propagation cannot be ignored, we also consider the permeability of the upper and lower adjacent layers based on two-point-flux approximation, which was discussed in details in our previous work^29^.

On the contrary, rock porosity ($\phi$) is a type of grid cell-based variable contributing to the fluid storage or accumulation term (first term) in Eq. (1), so we can also slice 3D porosity volumes into 2D horizontal layer-wise images without the need to consider vertical connectivity.

Next, the fluid phase rates $q_{\alpha }$ are functions of time, and also control the fluid source or sink term in Eq. (1). Since in a typical reservoir there are only a limited number of fluid injection or production wells, the 2D feature image of fluid phase rates is filled with the injection rate $q_{\alpha }$ at the location where the wells are drilled, yet zeros elsewhere.

Finally, a feature image of time *t* is incorporated to capture the temporal evolution of the state variable pressure (*p*), and to interpolate at intermediate time steps where no training data is available.

As we consider strong heterogeneity, e.g., the permeability *K* ranges from $10^{-4}$ to $10^4$ *milliDarcy* in our work. In order to reduce the permeability range, we scale it through natural logarithm. Therefore, the features to predict the state variable pressure (*p*) includes logarithmic permeability *log*(*K*), porosity $\phi$, fluid phase rate *q* and time *t*. All these features are further normalized between 0 and 1 through MinMax normalization. The details about how to assemble the feature and state variables are presented in the “Appendix” in Sect. 6.

### Feature coarsening

The asssembly of the 2D feature images as a feature array consumes large amount of computer memory, which can potentially lead to memory allocation issue (or error) and remarkably low training efficiency. Therefore, we coarsen the image size before proceeding with the training process. The feature images are coarsened by selecting spatial points with a constant stride, which is defined as an increment value added to the preceding spatial point in order to generate the next spatial point, while we always honor the most representative information, such as high permeability and porosity zones and injection well locations. Correspondingly, the output image of pressure is also processed in the same way.

**Figure 1:**
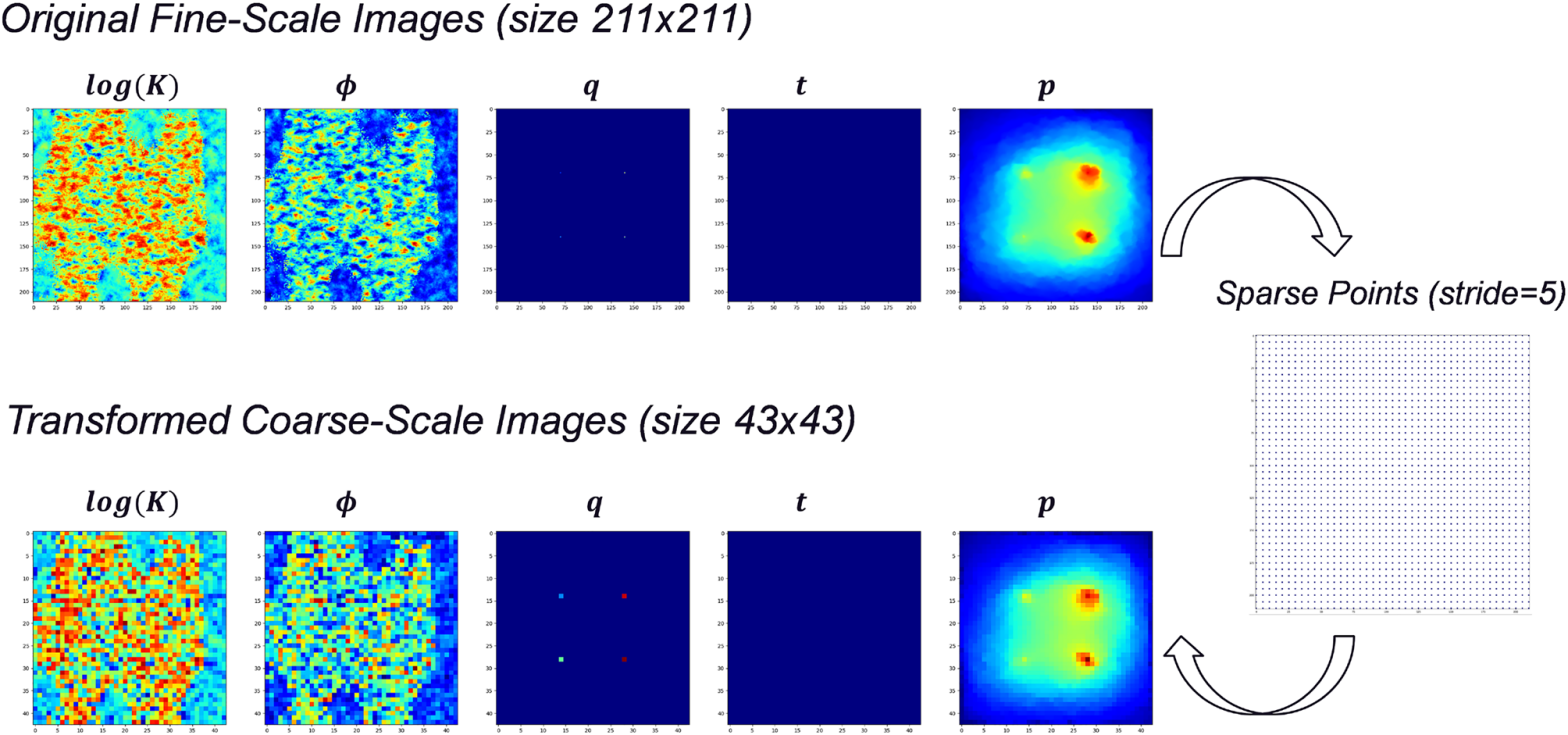
Transformed images of feature and state variables from fine-scale to coarse-scale.

An example of coarsening process is depicted in Fig. 1. By taking a stride of 5, the fine-scale images of feature and state variables of size $211\times 211$ are transformed into coarse-scale images of size $43\times 43$. During this process, the most representative information is well preserved in the coarsened images. Particularly, regions with warm colors in Fig. 1 represent the high permeability and high porosity zones, the nonzero injection rate values at the well locations and the pressure plume, and their shapes or spatial distributions are effectively captured at the coarse scale.

Table 1 shows the memory consumption of feature array changes with the feature image size as we perform the feature coarsening in Fig. 1. Notice here the feature array dimension equals to number of samples $\times$ image width $\times$ image length $\times$ number of feature types, and here the image width and length are consistent with the number of grid cells along the *x* and *y* directions in the reservoir model. The feature arrays are saved in float-32 (single) precision consistently to reduce redundant memory usage. It demonstrates that as we increase the stride from 1 to 10, the size of the 2D images decreases from $211\times 211$ to $22\times 22$, and the corresponding memory allocated to the feature array decreases by about 2 orders of magnitude (from 53.50 gigabytes to 0.58 gigabytes). The sensitivity analysis in Table 1 clearly demonstrates that the feature coarsening strategy significantly saves memory consumption.

**Table 1:** Sensitivity of stride with memory consumed by the feature array.

FNO stride	Image size	Array dimension	Memory (*M*), Gigabytes	Normalized *M*, $\%$
1	$211\times 211$	$80640\times 211\times 211\times 4$	53.50	$100\%$
2	$106\times 106$	$80640\times 106\times 106\times 4$	13.50	$25.24\%$
3	$73\times 73$	$80640\times 73\times 73\times 4$	6.40	$11.97\%$
4	$55\times 55$	$80640\times 55\times 55\times 4$	3.64	$6.79\%$
5	$43\times 43$	$80640\times 43\times 43\times 4$	2.22	$4.15\%$
10	$22\times 22$	$80640\times 22\times 22\times 4$	0.58	$1.09\%$

### Deep neural network (DNN) and training process

As we tackle large-scale heterogeneous geological models, an image-based approach is preferred to regress the high dimensional problem. The DNN we adopted in this work is the Fourier Neural Operator (FNO) proposed by^27^, which is demonstrated to excel in prediction for complex physics-based processes^24^. In FNO, the feature array *X* is first mapped into a high dimensional representation $V_0$ with 32 channels through a fully connected dense layer without applying activation function, shown as,

2 $$\begin{aligned} V_0 = W_0 \cdot X + b_0 \end{aligned}$$

Next, $V_l$ is recursively updated through Eq. (3),

3 $$\begin{aligned} V_l = \sigma (WV_{l-1} + \kappa (V_{l-1})), l=1, \dots , L. \end{aligned}$$

where $V_l$ are the feature maps at layer *l*, and is a function of $V_{l-1}$ preceding it; $\sigma$ denotes the nonlinear activation function; *W* is a linear operator defined by a 1D convolutional operator; $\kappa$ is a 2D convolution operator defined in the Fourier space. Ultimately, $V_L$ is transformed back to the state variable *p* through several fully connected dense layers.

Further, the feature maps is further propagate to even higher dimensional representation $V_{L+1}$ through a fully connected dense layer with applying activation function, shown as Eq. (4). Ultimately, $V_{L+1}$ is transformed back to the output layer with 1 channel through a fully dense layer without activation function, similar to Eq. (2).

4 $$\begin{aligned} V_{L+1} = \sigma (W_{L+1} \cdot V_{L} + b_{L+1}) \end{aligned}$$

**Figure 2:**
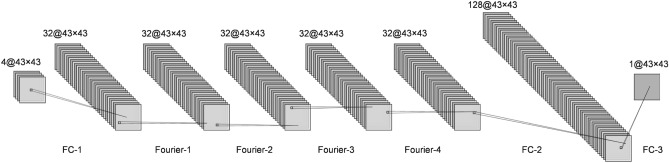
Architecture of Fourier Neural Operator with 3 fully connected (FC) and 4 Fourier layers. $\alpha @n_x \times n_y$ at the top of each layer denotes: *a* - number of features; $n_x$ - the image width; $n_y$ - the image length.

The architecture of FNO is shown in Fig. 2. To simplify the illustration, layers related to the operation in Eq. (3) are labeled as a “Fourier” layer, and fully connected dense layers based on Eqs. (2) and (4) are denoted as a “FC” layer. FNO takes 4 feature images ($log(K), \phi , q, t$) at layer FC-1, then sequentially goes through 4 Fourier layers and 2 FC layers to predict the output image of state variable *p*.

In Fig. 2, there is no activation function $\sigma$ involved in FC-1 and FC-3 layers, and the activation $\sigma$ used through Fourier-1 to Fourier-4 is a *LeakyReLU* defined as Eq. (5).

5 $$\begin{aligned} \sigma (x) = \left\{ \begin{array}{ c l } x &{} \quad \text {if } x \ge 0 \\ 0.01 &{} \quad \text {otherwise} \end{array} \right. \end{aligned}$$

The activation $\sigma$ adopted in FC-2 is *ReLU* defined as Eq. (6),

6 $$\begin{aligned} \sigma (x) = \left\{ \begin{array}{ c l } x &{} \quad \text {if } x \ge 0 \\ 0.0 &{} \quad \text {otherwise} \end{array} \right. \end{aligned}$$

Different from other convolutional neural networks, there is no pooling layer in FNO for down sampling and summarizing the average or the most activated presence of the feature maps. The loss function ${\mathcal {L}}$ in our FNO approach is defined as Eq. (7),

7 $$\begin{aligned} {\mathcal {L}}(\theta ) = ||\vec {p}-\vec {\hat{p}}|| + \lambda \cdot ||p_w - \hat{p}_w|| \end{aligned}$$

where $\theta$ are the learnable parameters; $||\cdot ||$ is the operator of root-mean-square-error (RMSE); $\vec {p}$ is the ground truth of the pressure field; $\vec {\hat{p}}$ is the prediction of $\vec {p}$ by FNO; $\lambda$ is a weighting factor; $p_w$ is the ground truth of *p* at the well locations; $\hat{p}_w$ is the prediction of $p_w$ by FNO. The second term in Eq. (7) is a regularization term for enhancing the resolution at the well locations.

8 $$\begin{aligned} \theta ^* = \mathop {\mathrm {argmin}}\limits _{\theta } {\mathcal {L}}(\theta ) \end{aligned}$$

The goal of training FNO is to find $\theta ^*$ by minimizing the loss function ${\mathcal {L}}$, as shown in Eq. (8). We implemented FNO and the associated training module with the deep learning library PyTorch^30^, and adopted the Adam optimizer to train FNO.

### Prediction resolution at fine scale

FNO performs prediction at the coarse scale, but in tasks like data assimilation^31,32^, we need to predict pressures at monitoring wells whose spatial locations are defined at the original fine scale coordinates but may not coincide with the coarser scale coordinates. The fine scale predictions are performed via spatial interpolation. During feature coarsening, we select the spatial points with a pre-defined stride, so the coarse-scale spatial coordinates $(x^c,y^c)$ can be easily tracked based on the fine-scale spatial coordinates $(x^f,y^f)$ and the stride, where superscripts *f* and *c* denotes fine and coarse scales, respectively. Assuming that the pressure $p^f$ at the fine scale is spatially continuous in the whole reservoir, $p^f$ can be recovered by interpolation based on the predicted pressure $p^c$ by FNO at the coarse scale and the coordinates $(x^c,y^c)$ and $(x^f,y^f)$, shown as Eq. (9),

9 $$\begin{aligned} p^f = {\mathcal {F}}(p^c, x^c, y^c, x^f, y^f) \end{aligned}$$

where ${\mathcal {F}}$ is a spatial interpolation operator. The interpolation scheme adopted by this work is the 2D piecewise cubic interpolation^33^. The hypothesis of pressure continuity in the whole reservoir holds well in most cases, except in scenarios that there is a no-flow boundary such as a sealing fault traversing the geological model, which induces local pressure discontinuity nearby the boundary. However, this type of scenario is not in the scope of this work.

We also remark that in multi-phase porous flow like $CO_2$ injection into saline aquifer, the evolution of the pressure plume is often much faster than that of the saturation plume^34^. Especially when there is not water production well for active pressure management, the $CO_2$ plume will not move far away from the injection well vicinity, due to the slightly compressible water phase. As a result, compared to pressure evolution, the evolution of $CO_2$ saturation is not necessary a global reservoir behavior. Since the fine-scale resolution recovery relies on the global continuity of the predicted state variable, this makes saturation not quite suitable to be predicted by the proposed methodology, if saturation plume is not globally distributed.

**Figure 3:**
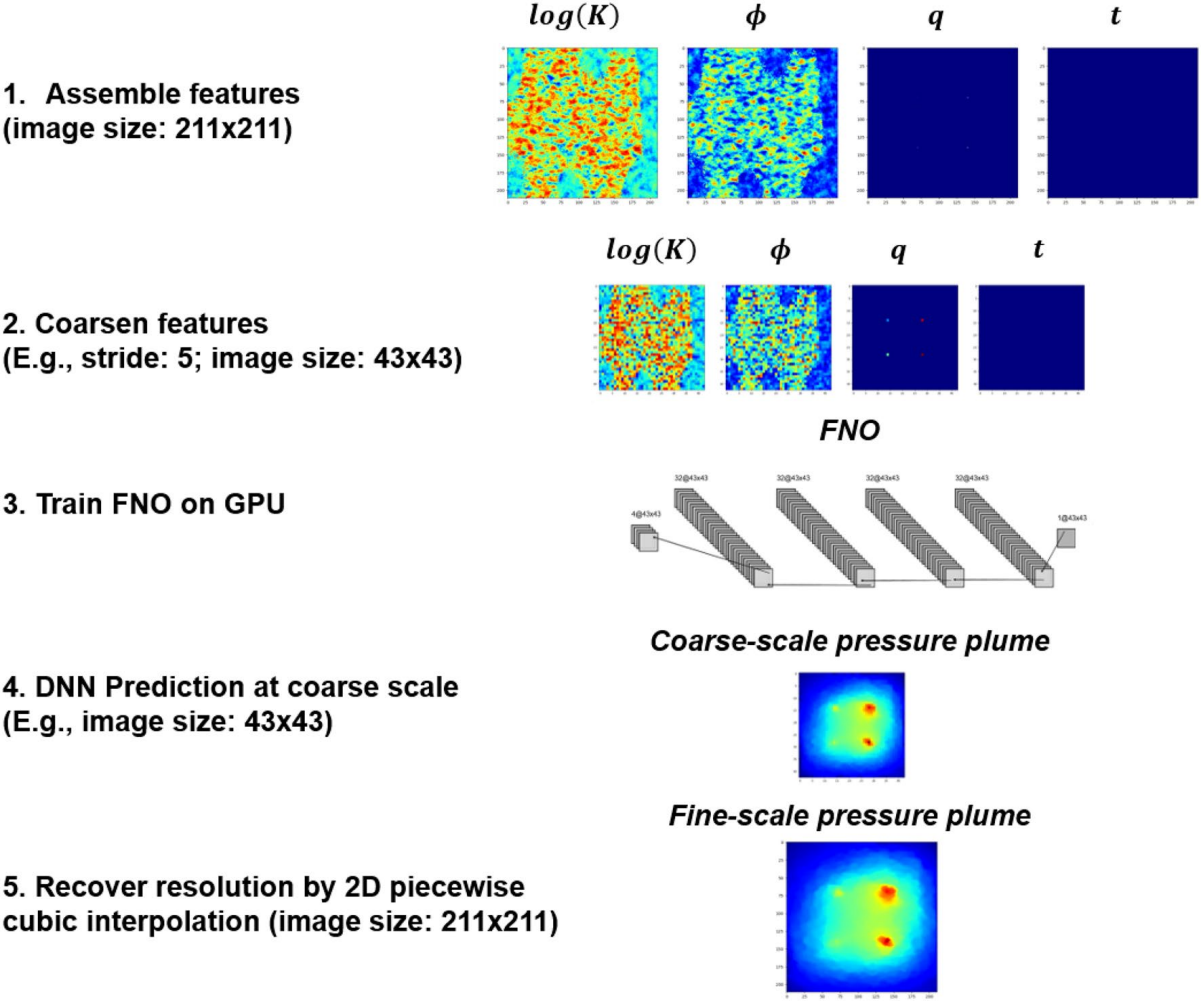
Deep Learning Workflow based on FNO considering feature coarsening and resolution recovery.

In a nutshell, the whole deep learning workflow is presented in Fig. 3, which includes 5 different steps as discussed above. In conventional deep learning workflow, there are only steps 1, 3 and 4 in Fig. 3. In this scenario, our training step cannot proceed due to memory shortage (total memory consumption of feature array: 53.50 gigabytes), unless we manually de-allocate some memories. As the DNN prediction is calculated in a coarse scale (step 4), the resolution recovery in step 5 becomes necessary in order to recover the full resolution. As we treat each individual horizontal reservoir layer as a single sample to FNO, the full 3D prediction of pressure field iterates through the prediction of each reservoir layer at each time step.

## Numerical experiments

### Reservoir model description

We use a 3D heterogeneous reservoir model with $211\times 211\times 30$ grid cells in the *x*, *y* and *z* directions respectively, resulting in 1335630 grid cells in total. The grid cell size is $500\times 500\times 10$ $ft^3$ in the *x*, *y* and *z* directions respectively, and is uniform throughout the model domain.

**Figure 4:**
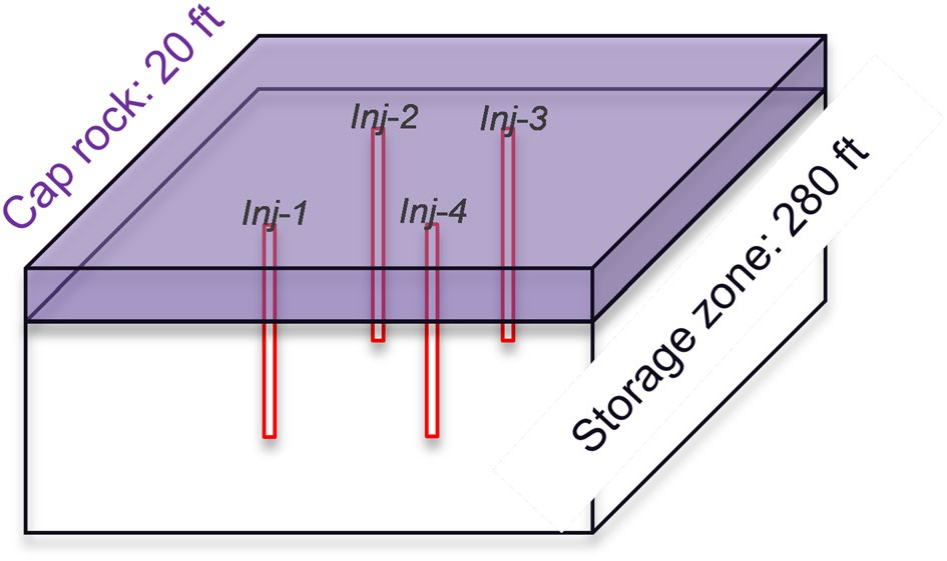
Schematic of the reservoir model for geological storage of $CO_2$.

The schematic of the reservoir model is shown in Fig. 4. There are 2 “caprock” layers (layers 1 and 2) to seal $CO_2$ at the top, and the “storage zone” is made up of 28 layers (layer 3 to 30) to store $CO_2$. There are 4 $CO_2$ injection wells perforated and injecting in all the 28 layers in the “storage zone”, and in the $x-y$ plane they are drilled at grid cell indices (71, 71), (71, 141), (141, 141) and (141, 71), respectively. Approximately $2\times 10^6$ *tons*/*year* of $CO_2$ was continuously injected into the reservoir for 10 years. The simulation results were reported and saved in 32 time steps, including monthly resolution at the beginning 2 years and yearly resolution from year 3 to 10.

**Figure 5:**
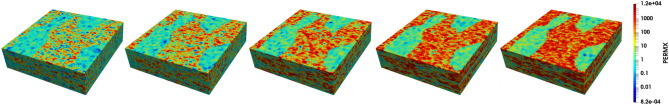
Realizations of permeability of the storage zone.

**Figure 6:**
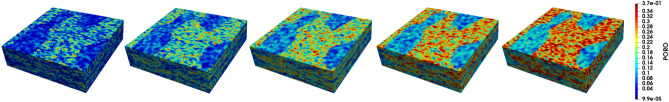
Realizations of porosity of the storage zone.

The permeability and porosity fields are correlated to each other. 100 equiprobable realizations of permeability and porosity fields are generated following the same geological facies model. Figure 5 presents the present the permeability realizations of the storage zone (left to right: P10, P25, P50, P75, P90), and the corresponding porosity realizations are presented in Fig. 6. In each example the shapes of the high permeability and porosity zones are quite similar, following the distribution of the rock facies, but the magnitudes of permeaility and porosity in these zones vary among different realizations.

Numerical simulations of $CO_2$ injection were performed with each of these 100 realizations using the commercial reservoir simulator, GEM by CMG^35^ in order to generate training data for the DL workflow. The average CPU time per simulation run is about 168.75 *seconds*. The ensemble of realizations is split based on the permeability and porosity realizations where we choose 90 simulation runs for training ($90\%$), 5 runs for validation ($5\%$) and 5 runs for testing ($5\%$).

### Training and prediction efficiency with feature coarsening

The remarkable reduction of memory consumption due to feature coarsening has been demonstrated in in Table 1. In this subsection, we further investigate the impact of feature coarsening on training and prediction efficiency. Different strides were chosen to coarsen the feature images and feed them to train the FNO model. The training samples are divided into mini-batches with 20 samples/batch, and we train the FNO models with 100 epochs on a GPU (NVIDIA Quadro RTX 4000), and weight factor in Eq. (7) is set to 0.1 based on our sensitivity analysis.

**Table 2:** Sensitivity of FNO stride with training and prediction efficiency.

FNO stride	Training CPU, h	Prediction CPU, s	Speedup to CMG
1	40.23	0.023	7337
2	10.47	0.24	703
3	9.02	0.12	1406
4	8.78	0.076	2220
5	8.86	0.066	2557
10	8.36	0.041	4116
CMG	–	168.75	-

Table 2 illustrates the training and prediction efficiency with different strides to coarsen the feature image size. Training at the full image size ($211\times 211$) requires 40.23 hours of CPU cost. As we coarsen the image by increasing the stride value, the training CPU time decreases by $74\%$ (10.47 hours for stride = 2) or $79\%$ (8.36 hours for stride = 10). This is primarily because smaller size images require less computation during the back-propagation process of training FNO. Therefore, the feature coarsening makes the training process much more affordable as we are dealing with a large scale geological model. The prediction includes FNO prediction and resolution recovery by spatial interpolation, except when stride is 1, where FNO predicts at the original fine scale and interpolation is not necessary. Overall, the prediction time decreases as we coarsen the feature image. We calculate the prediction speedup of DL compared to CMG GEM. The speedup compared to the CPU time needed for CMG’s GEM simulation run is remarkable, varying from 703 times (stride = 2) to 7337 times (stride = 1).

### Prediction accuracy

To measure the pressure relative error at each time step or each layer for the DL prediction, we calcualte the relative error of pressure based on $L-2$ norm, shown as,

10 $$\begin{aligned} e_p = \frac{||\vec {p}_i - \hat{\vec {p}}_i||_2}{||\vec {p}_i||_2} \times 100\% \end{aligned}$$

Where $i = 1, \dots , n_{ts}$ for the relative error at each timestep, with total number of time steps $n_{ts}=33$; or $i = 3, \dots , n_{z}$ for the relative error at each storage layer, with total number of storage layers $n_z = 30$; $\vec {p}_i$ is the pressure field of all cases at $i^{th}$ time step or layer predicted by CMG; $\hat{\vec {p}}_i$ is the pressure field of all cases at $i^{th}$ time step or layer predicted by DL;

**Figure 7:**
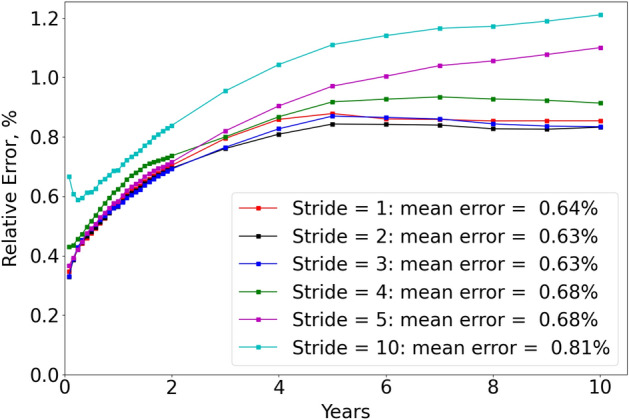
Pressure relative error with time under different strides based on the testing cases.

In Fig. 7, we plot the pressure relative error with time for different strides used for coarsening. It demonstrates that as the stride is increased to coarsen the feature images, the temporal error generally increases, and FNO with stride = 10 (cyan curve) has the highest temporal error (mean relative error $=0.81\%$). This can be explained by the fact that a coarsened image with larger stride will lose more information in the feature images and thus will lose more prediction fidelity. On the other hand, FNO with stride = 1 (red curve) that predicts at the original fine scale does not actually lead to the highest accuracy, which is likely due to the fact that the original fine-scale feature images, specifically permeability and porosity images, provide the FNO model with more information than necessary and make it less generalized as the scenarios with slightly coarsened resolution (e.g. stride = 2, 3).

**Figure 8:**
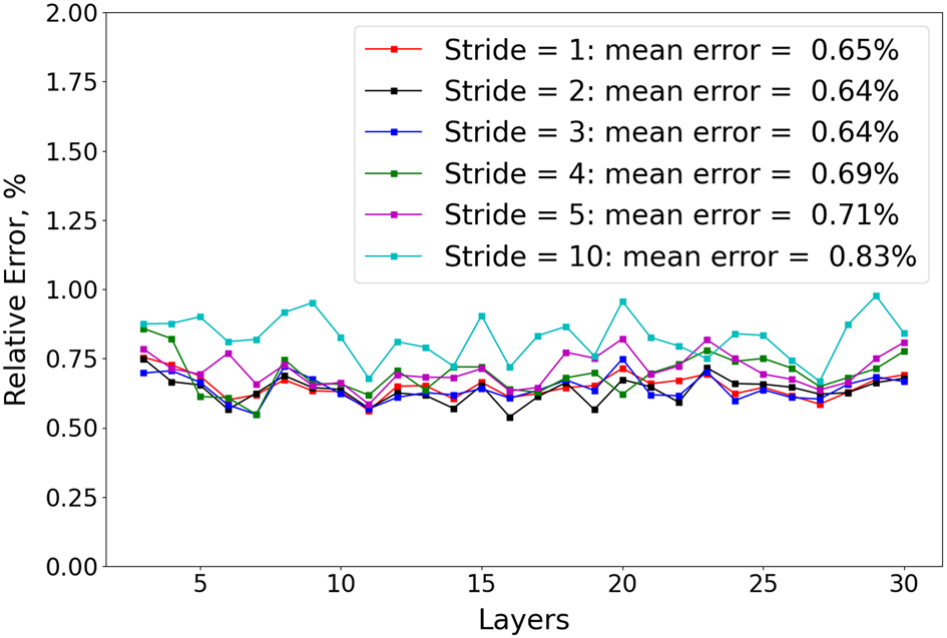
Pressure relative error with storage layers based on the testing cases.

We also plot the pressure relative error with different storage layers (from layer 3–30) under different strides, and the results are shown in Fig. 8. For FNO with different strides, we consistently observe that the prediction accuracy is quite stable in different layers. FNO with stride = 2 and 3 show the lowest mean relative errors. The stable performance of DL to different storage layers gives confidence to use the DL workflow to predict the pressure evolution in 3D.

Ultimately FNO with stride = 2, 3 brings the highest prediction accuracy, with mean relative error $=0.63\%$ among all time steps (Fig. 7) and mean relative error among all storage layers $=0.64\%$ (Fig. 8). However, FNO with stride = 2 takes longer time (10.47 hours) for training than FNO with stride = 3 (9.02 hours). Therefore, FNO with stride = 2 strikes the best balance between prediction accuracy and training efficiency. Besides, it also makes the prediction with a speedup of 1406 compared to physics-based reservoir simulator based on Table 2.

**Figure 9:**
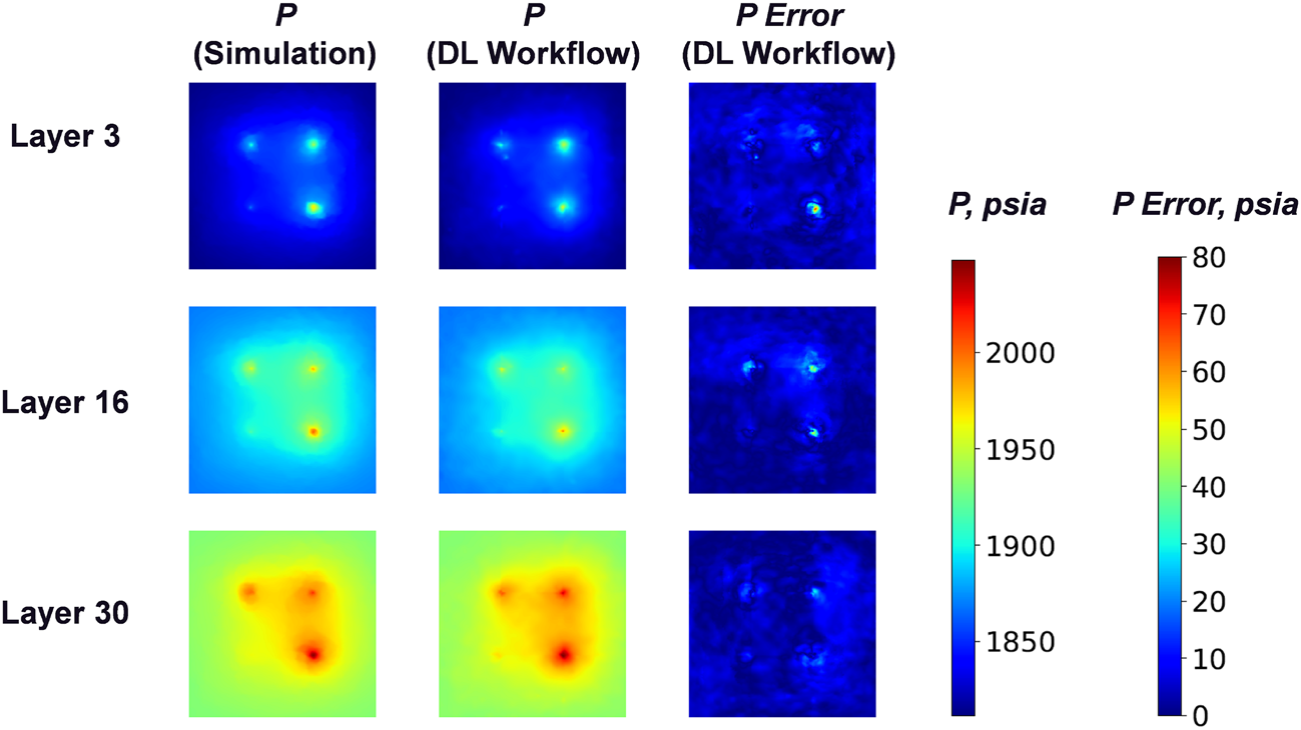
Pressure after 1 year injection. Mean absolute error: 2.826 *psia*, mean relative error: $0.149\%$.

**Figure 10:**
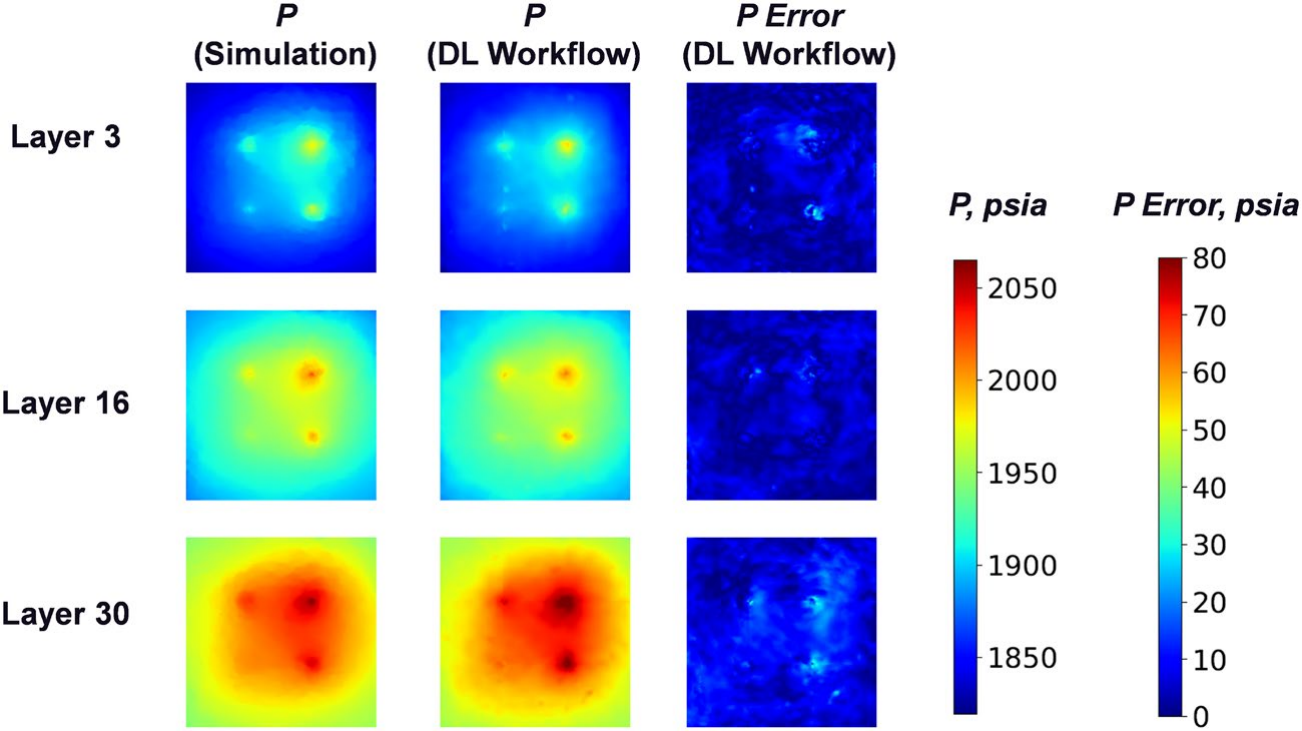
Pressure after 10 year injection. Mean absolute error: 3.906 *psia*, mean relative error: $0.202\%$.

In Figs. 9 and 10, we present a representative example of the pressure fields at different time steps in the top (layer 3), middle (layer 16) and bottom (layer 30) layers in the “storage zone” for predictions of the DL workflow with FNO (stride = 3), and compare them with the ground truth from physics-based reservoir simulation by CMG^35^. In Fig. 9, at the early period (1 year) the pressure plumes grow surrounding the 4 injection wells, and the plume sizes nearby different injection wells at layer 3 vary due to the difference of injectivity caused by permeability heterogeneity. Besides, the plume becomes larger with increasing depth (larger layer number) because of gravity. These details are captured by the predictions of our DL workflow (mean absolute error 2.826 *psia* and mean relative error $0.149\%$), and there are only small errors in the injection well locations. In Fig. 10, at the end of the $CO_2$ injection period (10 years) the pressure plume reaches its maximum size with the pressure error increasing slightly (mean absolute error 3.906 *psia* and mean relative error $0.202\%$). Besides, we observe that the DL workflow can generally predict smooth pressure field and delineates the irregular pressure plume shape driven by permeability and porosity heterogeneity quite well, which is benefited from the fine-scale resolution recovery by spatial interpolation based on the hypothesis of pressure continuity.

**Figure 11:**
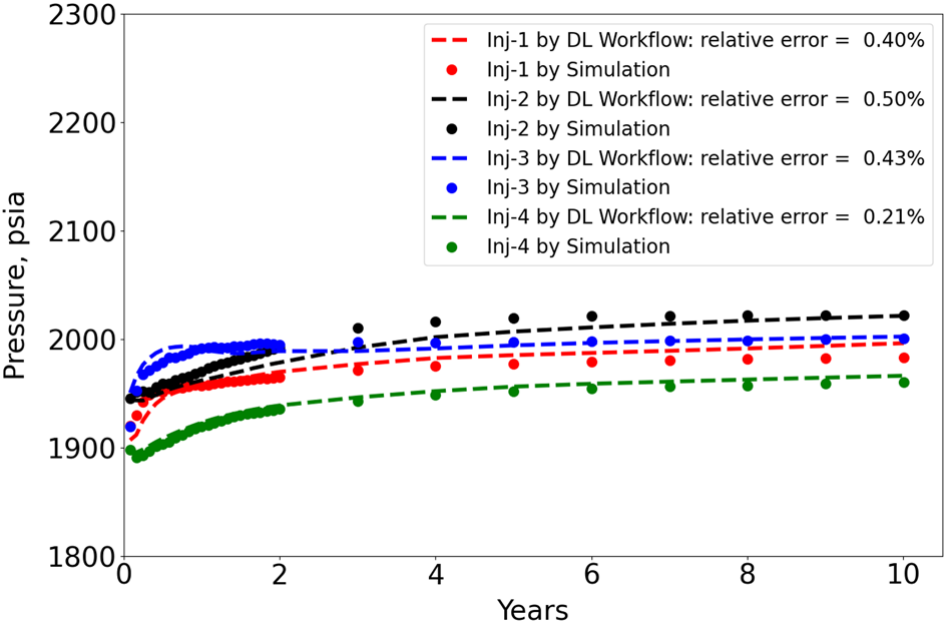
Pressure in the 4 injection well grid cells at layer 16 versus time.

**Figure 12:**
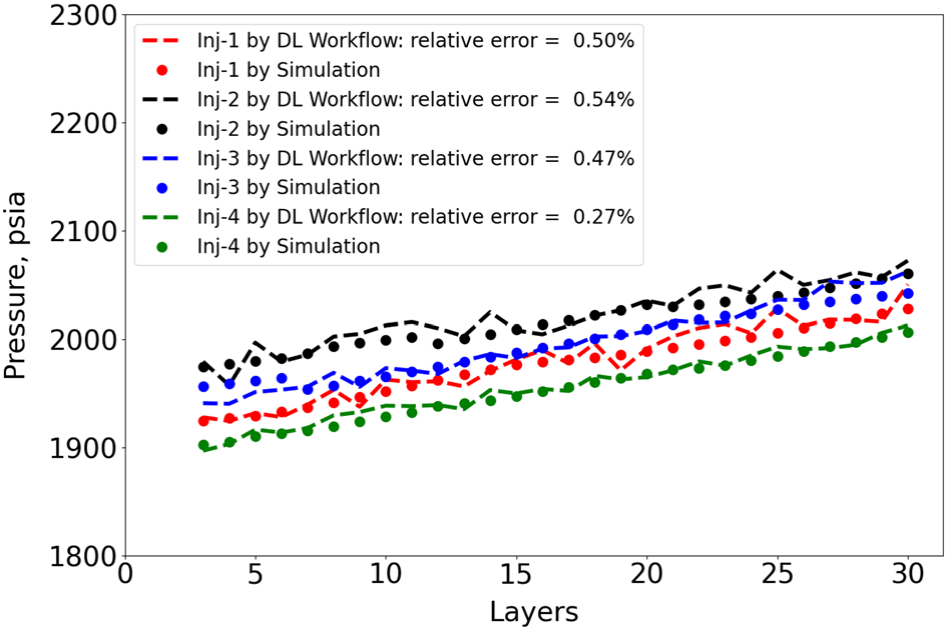
Pressure in injection well grid cells at year 10 versus storage layers.

In Fig. 11, we plot the pressure in the four injection well locations versus time. Notice that here we only choose the well grid at the middle storage layer (layer 16), and the well locations can be referred to Fig. 4. In the 10 years of $CO_2$ injection, the pressure in the well grid cells is relatively stable, with about 100 *psia* changes. The prediction by DL workflow (FNO with stride = 3) is quite close to CMG simulation, and the maximum relatively error is about $0.50\%$. In Fig. 12, we plot the pressure in the four injection well locations versus different layers at 10 years, and this helps to observe the prediction accuracy of DL at different vertical depths. As the reservoir depth increases with increasing layer number, we clearly see a monotonically increasing trend of well grid pressure. This is because of gravity, similar to what we observe in Figs. 9 and 10. The physics is accurately captured in the DL Workflow (FNO with stride = 3) and very consistent to the ground truth of CMG simulation, with maximum relative error $0.54\%$. The high prediction accuracy in the well grid cells is essential, since ultimately such data can be valuable prediction data for history matching or reservoir model calibration, which can be the next phase of this work.

## Conclusions

In this work, we developed a deep learning (DL) workflow to predict the pressure evolution due to fluid injection in 3D large-scale heterogeneous porous media. With a pre-defined stride, we performed feature coarsening to extract the most representative information of geology and well controls, which can help reduce memory consumption of feature arrays and improve training efficiency. Further, we recovered the resolution of the predicted pressure field to the fine scale based on the hypothesis of pressure continuity for fluid flow in porous media.

We evaluated the overall performance of the proposed workflow by applying it to the problem of $CO_2$ injection into a large-scale 3D heterogeneous aquifer. We demonstrated that the feature coarsening strategy significantly reduces the memory consumption by $>75\%$ and decreases the training time by $>74\%$, due to the fact that smaller feature image takes less computational time for the back-propagation in the training process. The feature coarsening process results in some fidelity loss during the prediction. On the other hand, the model trained at the original scale does not lead to the highest accuracy, due to the fact that fine scale images provide the DL workflow with too detailed of information and lead to a loss of generalization. In 3D pressure prediction, we obtained good temporal stability with relative pressure error $0.63\%$ across different time steps, and we also achieved decent spatial stability across all the layers with the layer-wise mean relative error $0.64\%$. The DL workflow can delineate with pressure plume shape accurately with great smoothness, which is benefited from the fine-scale resolution recovery by spatial interpolation based on the hypothesis of pressure continuity. The speed of prediction by the DL workflow is 1406 times faster than that of physics-based simulation, which is quite favorable for optimization and uncertainty quantification in many applications including $CO_2$ sequestration where physics-based simulations are computationally expensive.

## Data Availability

The data used and/or analyzed during the current study available from the corresponding author on reasonable request.

## Appendix: Feature and state variables assembly

The input features of log permeability ($\log (K)$) and porosity ($\phi$) can be directly wrangled from the simulation data in terms of these 3-dimensional (3D) property fields, and further saved as a high dimensional array with the size of $n_{run} \times n_x \times n_y \times n_z$. Here $n_{run}$ denotes number of simulation runs, $n_x$, $n_y$ and $n_z$ represent the number of grid cells in the *x*, *y*, and *z* directions, respectively. The fluid phase rates $q_{\alpha }$, specifically $CO_2$ injection rate in our numerical example, are wrangled from the simulation data and saved in time series data with the size of $n_{run} \times n_{ts} \times n_{well}$. Here $n_{well}$ represents number of injection wells, and $n_{ts}$ denotes the number of time steps in reservoir simulation, which is fixed among all the simulation runs. On the other hand, the feature of time step *t* is an 1-dimensional array with the size of $n_{ts}$. The state variable, e.g., pressure *p*, is wrangled from the simulation output data and saved as a high dimensional array with the size of $n_{run} \times n_{ts} \times n_x \times n_y \times n_z$.

As we treat each storage layer per time step per simulation run as a single sample, the total number of samples $n_{sample} = n_{run} \times n_{ts} \times n_z$. Algorithm 1 below is a workflow to convert the input features and state variables from CMG simulations into the data structure required by the DL framework.
